# Comparative Evaluation of Flexural and Impact Strengths of Glass Fiber- and Titanium Dioxide Nanoparticle-Reinforced Polymethyl Methacrylate Denture Base Resin: An In Vitro Study

**DOI:** 10.7759/cureus.112737

**Published:** 2026-07-15

**Authors:** V Ponjayanthi, S Vishali, Suganya Subramanian, Claudia Peter, Jeevitha Mani, Thangadurai Maheswaran

**Affiliations:** 1 Prosthodontics, Priyadarshini Dental College and Hospital, Thiruvallur, IND; 2 Prosthodontics, Vivekanandha Dental College for Women, Namakkal, IND; 3 Prosthodontics, Rajas Dental College and Hospital, Tirunelveli, IND; 4 Prosthodontics, Nandha Dental College and Hospital, Erode, IND; 5 Oral and Maxillofacial Pathology, Adhiparasakthi Dental College and Hospital, Melmaruvathur, IND

**Keywords:** denture base resins, flexural strength, glass fiber, heat-cured acrylic resin, hybrid reinforcement, impact strength, polymethyl methacrylate, reinforced denture material, titanium dioxide nanoparticle

## Abstract

Objective: This in vitro study aimed to evaluate the effects of glass fiber and titanium dioxide (TiO₂) nanoparticle reinforcement on the flexural and impact strengths of polymethyl methacrylate (PMMA) denture base resin.

Methods: Forty rectangular heat-activated acrylic denture base specimens with dimensions of 65 mm × 10 mm × 3.3 mm and 80 mm × 4 mm × 10 mm were fabricated to determine the flexural and impact strengths in accordance with ISO#1567 with an estimated sample size of 10 per group, which was further divided into five for flexural and five for impact strength. Group 1 (Control group) contains conventional heat-activated acrylic denture base resin, Group 2 has 2% TiO_2_ nanoparticle-incorporated heat-cured acrylic denture base resin, Group 3 comprises 2% glass fiber-incorporated heat-cured acrylic denture base resin, and Group 4 (Hybrid group) comprises 2% TiO_2_+2% glass fiber-incorporated heat-cured acrylic denture base resin. The fabrication followed the compression molding technique and a long curing cycle, and the specimens were tested for flexural and impact strengths.

Result: The hybrid reinforcement group (Group 4; 2% TiO₂ + 2% glass fiber) demonstrated the highest flexural strength (100.32 ± 4.21 MPa), followed by the glass fiber-reinforced group (Group 3; 92.71 ± 3.93 MPa), the TiO₂-reinforced group (Group 2; 84.87 ± 3.78 MPa), and the conventional PMMA group (Group 1; 76.35 ± 2.61 MPa). Similarly, the highest impact strength was observed in Group 4 (5.13 ± 0.43 kJ/m²), followed by Group 3 (4.90 ± 0.39 kJ/m²), Group 2 (3.87 ± 0.45 kJ/m²), and Group 1 (1.48 ± 0.26 kJ/m²).

Conclusion: Hybrid reinforcement with TiO_2_ and glass fiber exhibited better flexural and impact strengths than conventional TiO_2_ and glass fiber-reinforced PMMA.

## Introduction

Polymethyl methacrylate (PMMA) is mostly used in the fabrication of permanent denture bases because of its exceptional properties and biocompatibility, with the exception of its shortcomings with respect to its mechanical properties, including low resistance to impact and flexural stresses [1,2]. Most maxillary denture fractures result from fatigue and impact failure, whereas in the mandible, 80% of denture fractures result from impact failure [3,4]. Enhancing the material's strength characteristics can help avoid the need for repeated denture fabrication and multiple appointments, especially for elderly patients.

Various reinforcing strategies, including the use of nanoparticles, butadiene-styrene, metal wires, PMMA graft copolymers, and fibers such as aramid, carbon, polyethylene, glass, and polypropylene, have been reported in the literature [1-4]. Among all the materials, PMMA reinforced with nanoparticles and glass fibers has been preferred for its ability to improve strength, wear and tear resistance, and antimicrobial properties [5]. The characteristics of denture base resins are significantly affected by the surface area-to-volume ratio of the nanoparticles [6]. Among the available nanoparticles, titanium dioxide (TiO₂) nanoparticles remain one of the preferred alternatives because of their chemical stability, ease of availability, robust physical properties, low toxicity, cost-effectiveness, and antibacterial activity [7]. Glass fibers have good biological properties, better aesthetic appearance, better bonding with acrylic resin, and a high level of chemical resistance, and is comparatively cost-effective [8,9]. It can be easily manipulated and combined with polymer powders. Glass fiber reinforcement has been found to significantly increase the impact strength of PMMA denture base resin [10].

The clinical importance of enhancing denture base materials lies in reducing structural failures, thereby improving patient satisfaction and minimizing the need for repeated prosthesis replacement and clinical visits. Although individual reinforcement approaches have been well documented, the combined effect of glass fibers and TiO₂ nanoparticles within a hybrid PMMA matrix remains insufficiently explored. Accordingly, the present study aimed to evaluate and compare the effect of reinforcing heat-cured PMMA denture base resin with glass fibers and TiO₂ nanoparticles on its flexural and impact strengths. The null hypothesis stated that the combined incorporation of glass fibers and TiO₂ nanoparticles would not produce a statistically significant effect on the flexural and impact strengths of heat-cured PMMA denture base resin.

## Materials and methods

This study was conducted at the Department of Prosthodontics, Vivekanandha Dental College for Women, Namakkal, India, between December 2023 and February 2024. A total of 40 rectangular heat-cured acrylic denture base specimens of size 65 mm × 10 mm × 3.3 mm and 80 mm × 4 mm × 10 mm were fabricated to determine the flexural and impact strengths in accordance with ISO #1567, similar to the work by Mowade et al. [5] and Anjum et al. [11]. To ensure adequate statistical validity, the sample size for mechanical testing was determined prospectively using G*Power software (version 3.1.9.7; Heinrich-Heine-Universität Düsseldorf, Düsseldorf, Germany). The analysis was performed with a significance level (α) of 0.05 and a statistical power (1 - β) of 0.80. Based on a large anticipated effect size (f), estimated from pilot data and previous literature, the minimum required sample size was calculated as five specimens per group to detect statistically significant differences in flexural and impact strengths between the control and hybrid-reinforced groups.

Group 1 (control group): Conventional heat-activated acrylic denture base resin (DPI, India).

Group 2: 2wt% TiO₂ nanoparticle (Ultrananotech, India) incorporated into heat-cure acrylic denture base resin.

Group 3: 2 wt% glass fiber (DPI, India) incorporated into heat-activated acrylic denture base resin.

Group 4 (hybrid group): 2 wt% TiO₂ nanoparticles + 2 wt% glass fiber-reinforced heat-activated acrylic denture base resin.

**Figure 1:**
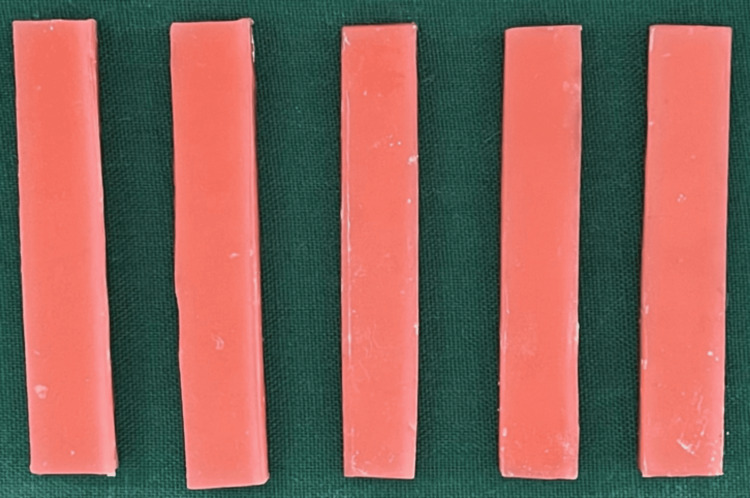
Rectangular wax model

**Figure 2:**
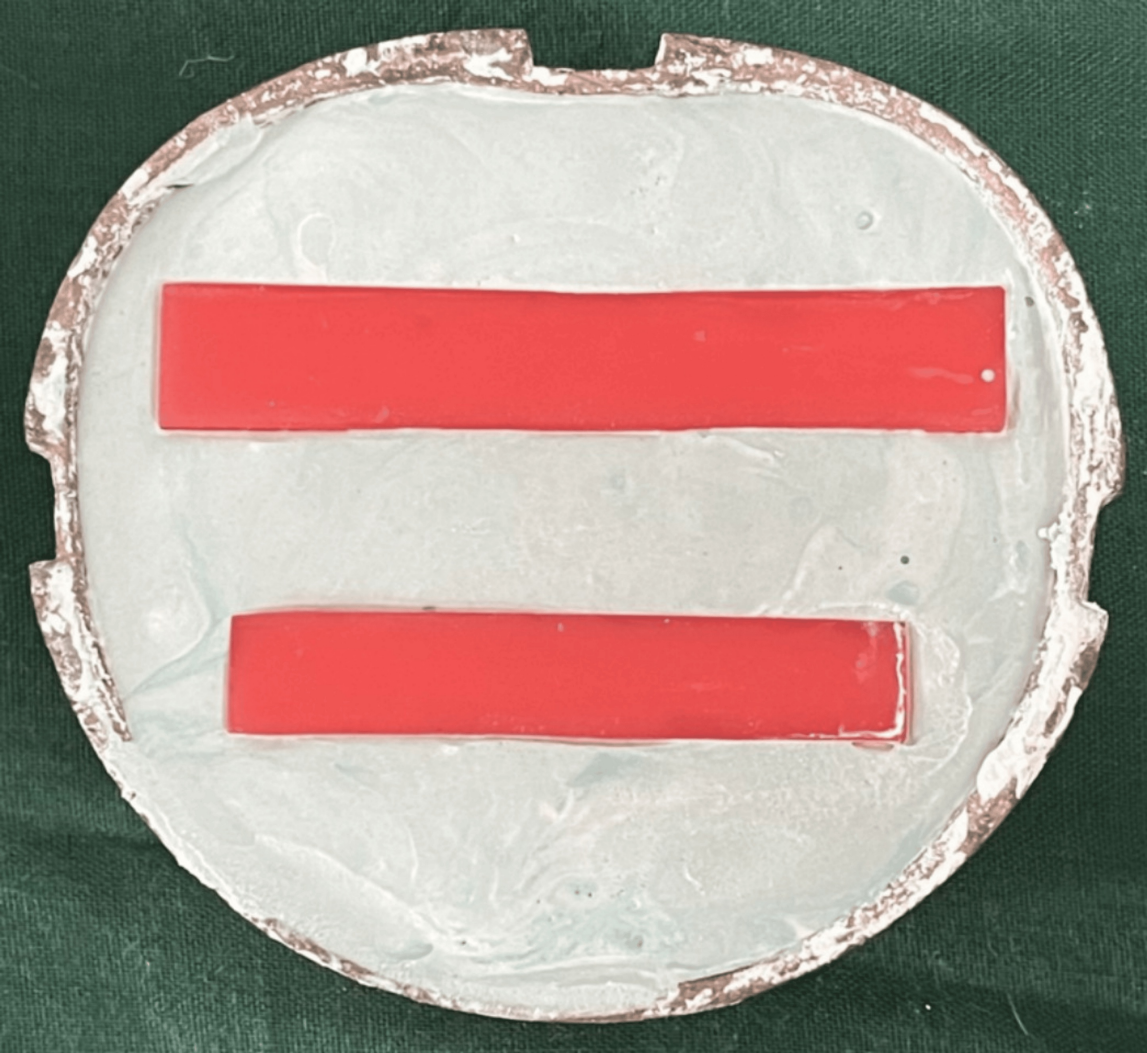
Creation of mold space

Initially, a rectangular wax model with internal dimensions of 65 mm × 10 mm × 3.3 mm and 80 mm × 4 mm × 10 mm was fabricated (Figure 1), and a mold space was created by dewaxing the model (Figure 2).

**Figure 3:**
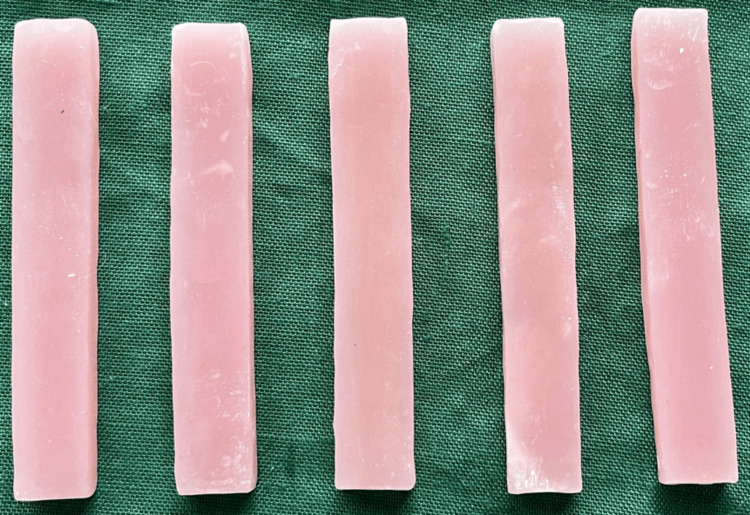
Group I heat cure samples for impact strength

**Figure 4:**
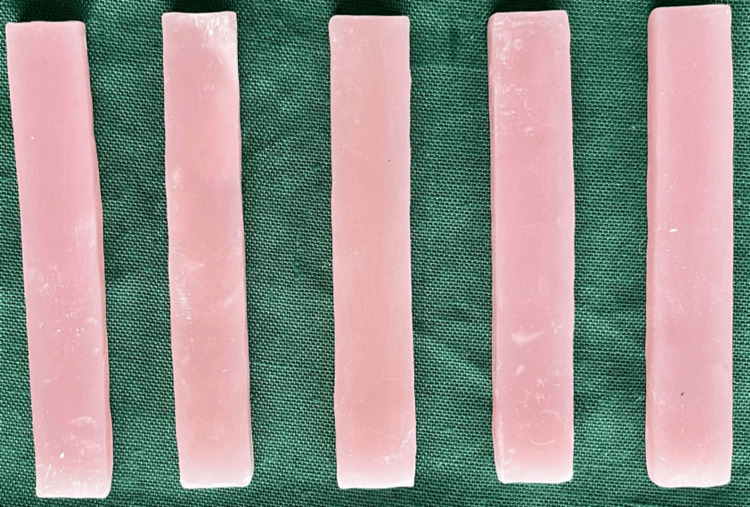
Group I heat cure samples for flexural strength

Group 1 was fabricated by mixing the heat-cured PMMA polymer with the heat-cured monomer, one set of five for impact strength assessment (Figure 3) and another set of five for flexural strength assessment (Figure 4).

**Figure 5:**
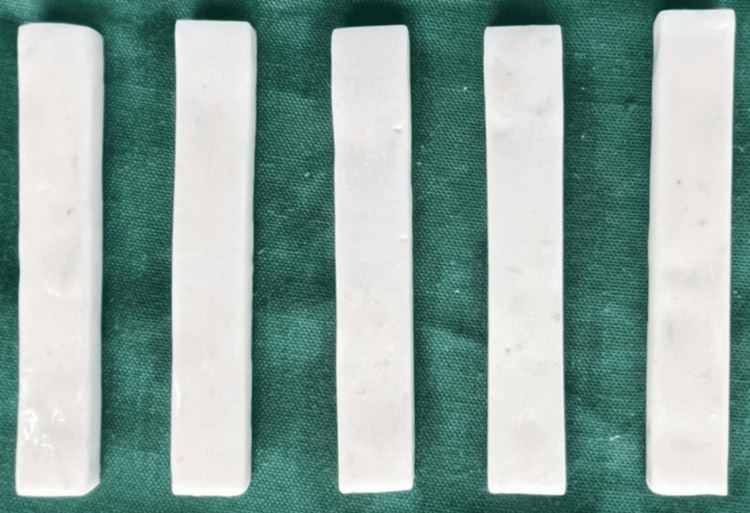
Group II 2% titanium dioxide-reinforced PMMA samples for impact strength PMMA: polymethyl methacrylate

**Figure 6:**
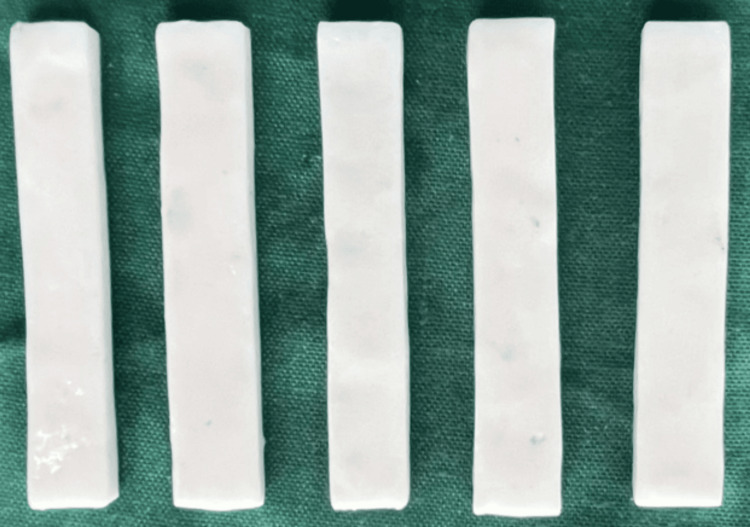
Group II 2% titanium dioxide-reinforced PMMA samples for flexural strength PMMA: polymethyl methacrylate

Group 2 was fabricated by incorporating 2 g of TiO₂ nanoparticles into 98 g of heat-cured polymer and mixing it with heat-cured monomer (Figure 5) for Impact and (Figure 6) for flexural strength assessment.

**Figure 7:**
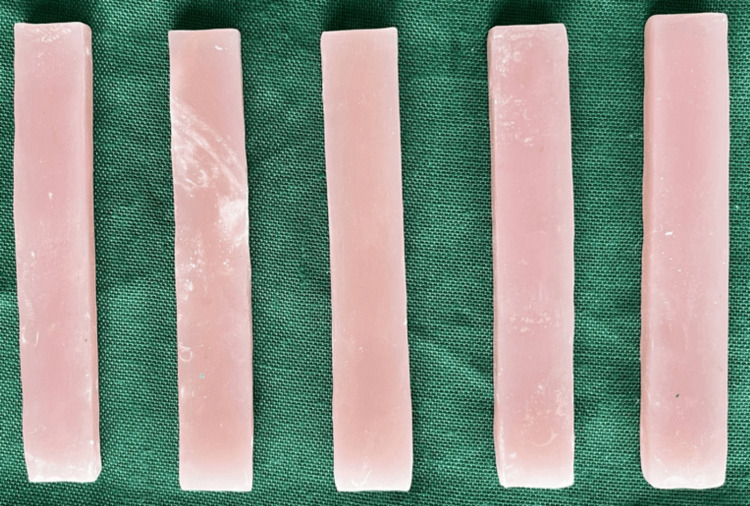
Group III 2% glass fiber-reinforced PMMA samples for impact strength PMMA: polymethyl methacrylate

**Figure 8:**
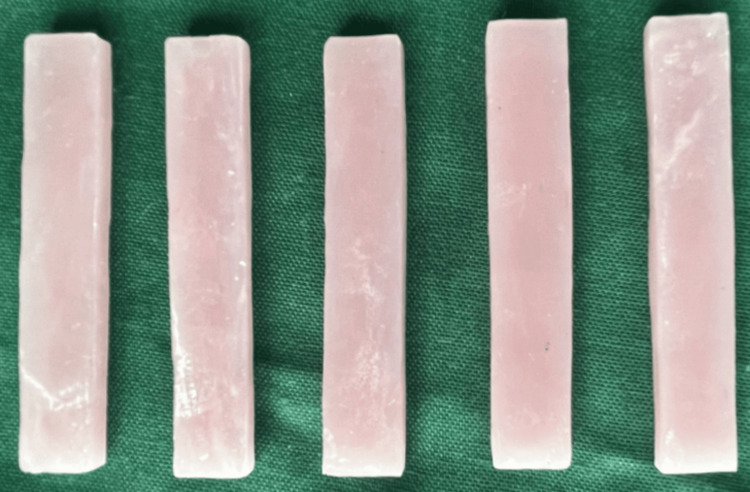
Group III 2% glass fiber-reinforced PMMA samples for flexural strength PMMA: polymethyl methacrylate

Group 3 was fabricated by adding 2 g of glass fiber reinforcement into 98 g of heat-cured polymer and mixing it with heat-cured monomer (Figure 7) for impact and (Figure 8) for flexural strength.

**Figure 9:**
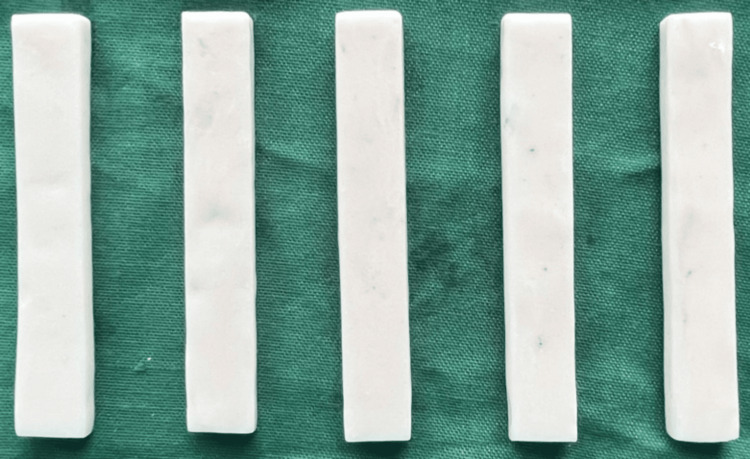
Group IV 2% titanium dioxide and 2% glass fiber-reinforced PMMA samples for impact strength PMMA: polymethyl methacrylate

**Figure 10:**
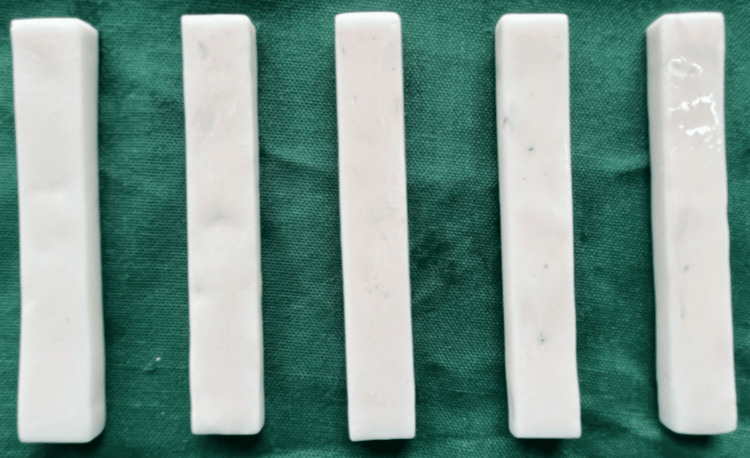
Group IV 2% titanium dioxide and 2% glass fiber-reinforced PMMA samples for flexural strength PMMA: polymethyl methacrylate

Group 4 was fabricated by adding 2 g of TiO₂ nanoparticles and 2 g of glass fiber reinforcement to 96 g of heat-cured polymer and mixing it with heat-cured monomer (Figures 9, 10). Processing was carried out using a conventional compression molding technique and a long curing cycle.

Testing for flexural strength

**Figure 11:**
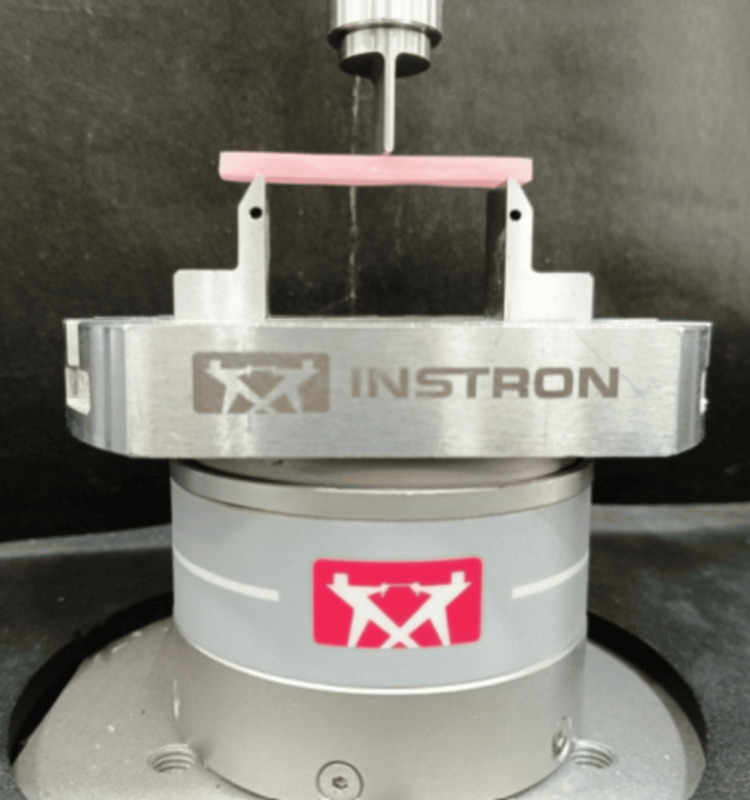
Transverse load applied to the sample

The specimens were subjected to a three-point bending test using an E3000 universal testing machine (Instron, Norwood, MA). A transverse load was applied to the specimens until fracture (Figure 11). The flexural strength of the specimens was measured in megapascals.

Testing for impact strength

**Figure 12:**
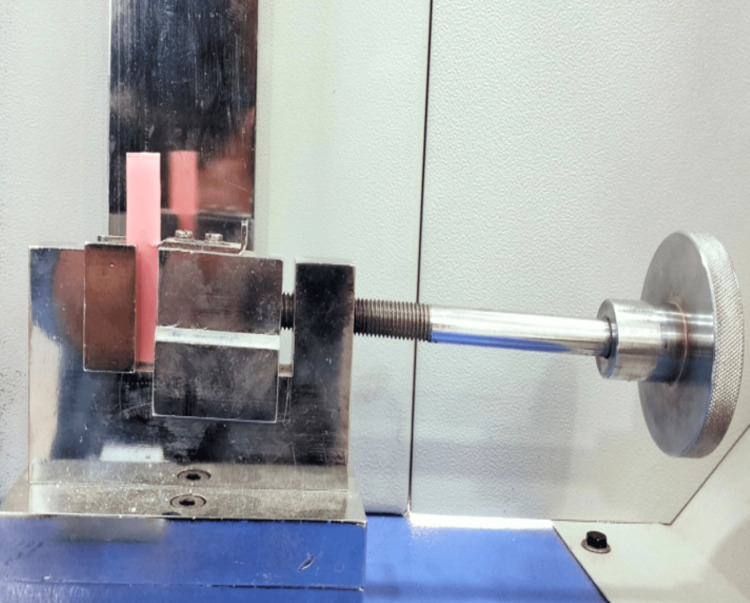
Impact load applied to the sample

Specimens measuring 80 mm × 10 mm × 4 mm were subjected to impact testing using a Charpy pendulum impact tester. Each specimen was positioned horizontally and loaded until fracture occurred (Figure 12). The impact strength was recorded in kilojoules per square meter.

Statistical analysis

The data obtained were analyzed using the IBM Statistical Package for Social Sciences software version 25.0 (IBM Corp., Armonk, NY). Descriptive statistics (mean and standard deviation) were obtained. The normality of the data was assessed, which revealed that the data were normally distributed. A comparison of all three groups was performed using one-way analysis of variance (ANOVA), followed by post hoc tests. The level of significance was set at 5% (p < 0.05 indicates statistical significance).

## Results

Flexural strength

The flexural strength values of each sample were obtained in megapascals, and the results were statistically analyzed. Table 1 summarizes the comparison of flexural strength among the four study groups, demonstrating a statistically significant difference, with the hybrid-reinforced PMMA group exhibiting the highest mean flexural strength. As shown in Table 2, post hoc analysis revealed significant pairwise differences in flexural strength, with the hybrid-reinforced PMMA group demonstrating superior performance compared with the other groups.

**Table 1:** Comparison of flexural strength among conventional PMMA, TiO₂-reinforced PMMA, glass fiber-reinforced PMMA, and hybrid-reinforced PMMA using one-way ANOVA Values are presented as mean ± SD. Flexural strength was measured in megapascals. Intergroup comparison was performed using ANOVA. A p value of <0.05 was considered statistically significant PMMA: polymethyl methacrylate, TiO₂: titanium dioxide, SD: standard deviation, ANOVA: analysis of variance

Groups	Sample size	Mean ± SD	F	p value
Control group	5	76.35 ± 2.61	39.078	<0.001
TiO₂-reinforced PMMA	5	84.9 ± 3.9		
Glass fiber-reinforced PMMA	5	92.7 ± 3.93		
TiO₂ and glass fiber-reinforced PMMA	5	100.32 ± 4.21		

**Table 2:** Pairwise comparison of flexural strength among study groups using post hoc analysis Pairwise comparisons of flexural strength between groups were performed using post hoc multiple comparison testing following one-way ANOVA. Mean difference represents the difference between Group I and Group J. A p value of <0.05 was considered statistically significant PMMA: polymethyl methacrylate, TiO₂: titanium dioxide, ANOVA: analysis of variance ^*^Statistically significant difference (p < 0.05)

Group (I)	Group (J)	Mean difference (I-J)	p value
Control group	TiO₂-reinforced PMMA	8.51800^*^	0.000
	Glass fiber-reinforced PMMA	-7.84600^*^	0.018
	TiO₂ and glass fiber-reinforced PMMA	-15.45400^*^	0.023
TiO₂-reinforced PMMA	Control group	23.97200^*^	0.000
	Glass fiber-reinforced PMMA	15.45400^*^	0.000
	TiO₂ and glass fiber-reinforced PMMA	7.60800^*^	0.023
Glass fiber-reinforced PMMA	Control group	-8.51800^*^	0.010
	TiO₂-reinforced PMMA	-16.36400^*^	0.018
	TiO₂ and glass fiber-reinforced PMMA	-23.97200^*^	0.000
TiO₂ and glass fiber-reinforced PMMA	Control group	16.36400^*^	0.010
	TiO₂-reinforced PMMA	7.84600^*^	0.000
	Glass fiber-reinforced PMMA	-7.60800^*^	0.000

Impact strength

Table 3 presents a comparison of impact strength among the four groups and shows a statistically significant increase in impact strength after reinforcement, particularly in the hybrid-reinforced PMMA group.

**Table 3:** Comparison of impact strength among conventional PMMA, TiO₂-reinforced PMMA, glass fiber-reinforced PMMA, and hybrid-reinforced PMMA using one-way ANOVA Values are expressed as mean ± SD. Impact strength was measured in kilojoules per square meter. Intergroup comparisons were performed using one-way ANOVA. A p value of <0.05 was considered statistically significant PMMA: polymethyl methacrylate, TiO₂: titanium dioxide, SD: standard deviation, ANOVA: analysis of variance

Groups	Mean ± SD	F	p value
Control group	1.4760 ± 0.26121	92.865	0.000
TiO₂-reinforced PMMA	3.8740 ± 0.44646		
Glass fiber-reinforced PMMA	4.8960 ± 0.38837		
TiO₂ and glass fiber-reinforced PMMA	5.1320 ± 0.42752		

As shown in Table 4, post hoc analysis revealed significant pairwise differences in impact strength across most study groups, with the hybrid-reinforced PMMA group exhibiting the greatest impact resistance.

**Table 4:** Pairwise comparison of impact strength among study groups using post hoc analysis Post hoc multiple comparison analysis was performed following one-way ANOVA to identify differences in impact strength between individual groups. Mean difference represents the difference between Group I and Group J. A p value of <0.05 was considered statistically significant PMMA: polymethyl methacrylate, TiO₂: titanium dioxide, ANOVA: analysis of variance ^*^Statistically significant difference (p < 0.05)

Group (I)	Group (J)	Mean difference (I-J)	p value
Control group	TiO₂-reinforced PMMA	3.42000^*^	0.000
	Glass fiber-reinforced PMMA	1.02200^*^	0.004
	TiO₂ and glass fiber-reinforced PMMA	-0.23600	0.772
TiO₂-reinforced PMMA	Control group	3.65600^*^	0.000
	Glass fiber-reinforced PMMA	1.25800^*^	0.001
	TiO₂ and glass fiber-reinforced PMMA	0.23600	0.772
Glass fiber-reinforced PMMA	Control group	2.39800^*^	0.000
	TiO₂-reinforced PMMA	-1.02200^*^	0.004
	TiO₂ and glass fiber-reinforced PMMA	-1.25800^*^	0.001
TiO₂ and glass fiber-reinforced PMMA	Control group	-2.39800^*^	0.000
	TiO₂-reinforced PMMA	-3.42000^*^	0.000
	Glass fiber-reinforced PMMA	-3.65600^*^	0.000

## Discussion

PMMA denture base resin is the most commonly used material for fabricating a complete denture. Its popularity and use stem from a combination of positive attributes rather than a single desirable property, including ease of processing, biocompatibility, stability in the oral environment, low cost, and acceptable esthetics [12]. While it meets esthetic demands, it falls short of fulfilling the mechanical requirements of prostheses. Impact failure from unintentional falls and flexural failure during mastication are the most prevalent causes of denture fractures. A study conducted by Johnston et al. reported that approximately 68% of acrylic denture bases experience breakage within a few years of fabrication, primarily due to impact failure [13,14]. According to Naik, 53% of lower denture fractures occur due to accidental falling of the denture while cleaning, removal, and insertion [15].

The primary approach to strengthening denture base polymers involves incorporating reinforcements such as metals, nanoparticles, and fibers. Among the different types of fibers, glass fiber stands out as the most widely utilized reinforcing fiber owing to its favorable mechanical properties and resistance to heat and chemicals [16]. It blends well with polymer powders and is easy to work with. The impact strength of the PMMA denture base resin was considerably increased by glass fiber reinforcement [10,17].

A lower concentration of 2% was employed in this investigation because glass fibers can cluster together, and the possibility of porosity increases when a higher amount of glass fiber is combined with the polymer.

Compared with other nanoparticles, titanium has been used in this study because its effectiveness in improving the mechanical properties of PMMA is primarily attributed to the size, shape, properties, and bonds formed between titanium oxide nanoparticles and PMMA [7,18]. It increases surface hydrophobicity, reduces biomolecule adherence, aids in coloring, and has antimicrobial properties [19]. In a study conducted by Ahmed et al., TiO₂ nanoparticles were incorporated into PMMA at concentrations of 1% and 5%. The findings revealed that adding TiO₂ nanoparticles at concentrations above 1% significantly increased the impact strength of the denture bases [20]. Therefore, a 2% percentage was chosen for this study.

This study employed a combination of 2% TiO₂ nanoparticles and 2% glass fibers, which possess commendable esthetic qualities and good strength. This study aimed to identify the effects of hybrid reinforcement and superior materials that could be utilized in future clinical applications, thereby enhancing prosthesis longevity and ensuring patient comfort.

Forty samples were fabricated, including 10 conventional heat-cured samples, 10 TiO₂ nanoparticle-reinforced PMMA samples, 10 glass fiber-reinforced PMMA samples, and 10 TiO₂ nanoparticle-and-glass fiber-reinforced PMMA samples. Samples with dimensions of 65 mm × 10 mm × 3.3 mm were subjected to a three-point bending test in a universal testing machine to determine the flexural strength.

The mean ± standard deviation values of the flexural strength for Groups 1, 2, 3, and 4 were 76.35 ± 2.61, 84.87 ± 3.78, 92.71 ± 3.93, and 100.32 ± 4.21 MPa, respectively. TiO₂ and glass fiber-reinforced PMMA denture base resins exhibited the highest flexural strength among all groups. Upon conducting an ANOVA, a statistically significant difference was revealed across all four groups. A post hoc test was performed to further understand the variations among the groups. The findings demonstrated that PMMA reinforced with both TiO₂ nanoparticles and glass fibers exhibited the highest flexural strength, followed by glass fiber-reinforced and TiO₂ nanoparticle-reinforced PMMA. The superior flexural strength observed in the hybrid reinforcement group may be attributed to the complementary reinforcing mechanisms of the glass fibers and TiO₂ nanoparticles. Glass fibers primarily act as stress-bearing components that bridge developing cracks and effectively transfer applied loads through the resin matrix, thereby delaying crack initiation and propagation [21,22]. In contrast, TiO₂ nanoparticles reinforce the polymer matrix at the microscopic level by restricting polymer chain mobility and improving stress distribution throughout the resin [23,24]. When incorporated together, these additives may produce a synergistic effect, resulting in greater resistance to bending stresses than either reinforcement used individually. The improved interaction among the resin matrix, fibers, and nanoparticles may also contribute to more efficient stress transfer under loading.

Subsequently, the addition of glass fiber reinforcement also enhanced the flexural strength of PMMA denture base resin. These findings are consistent with previous research on fiber reinforcement. Vallittu et al. demonstrated that novel glass fiber reinforcements significantly enhance the flexural properties of multiphase dental polymers, attributing this improvement to the proper impregnation of fibers within the polymer matrix [25]. Similarly, Kanie et al. evaluated the impact strength of a woven glass fiber-reinforced acrylic resin at various depths (0.5, 1.0, and 1.5 mm below the surface). Their results indicated that while glass fiber reinforcement generally improved impact strength, the greatest enhancement occurred when the fibers were positioned more superiorly (closer to the surface) [26]. Furthermore, the TiO₂-reinforced PMMA denture base resin exhibited higher flexural strength than neat PMMA, consistent with the results reported by Hernandez et al. [27]. Notably, the control group exhibited the lowest flexural strength, as observed in this study. Although most studies have reported improvements in the mechanical properties of PMMA following reinforcement with glass fibers or TiO₂ nanoparticles, the magnitude of improvement has varied considerably across investigations. Such variability may be attributed to differences in fiber length, fiber orientation, silane surface treatment, nanoparticle size, concentration, dispersion technique, polymerization cycle, specimen dimensions, and testing methodology [28]. The agglomeration of nanoparticles at higher concentrations can create stress concentration sites that reduce the mechanical performance, whereas inadequate impregnation or random distribution of glass fibers can compromise stress transfer within the resin matrix.

The mean ± standard deviation values of the impact strength for Groups 1, 2, 3, and 4 were 1.48 ± 0.26, 3.87 ± 0.45, 4.90 ± 0.39, and 5.13 ± 0.43, respectively. Notably, Group 4 exhibits a higher impact strength than the other groups. Furthermore, when conducting a post hoc test to compare impact strength between groups, the resulting order was as follows: TiO₂- and glass fiber-reinforced PMMA > glass fiber-reinforced PMMA > TiO₂-reinforced PMMA > conventional PMMA. The greater improvement in the impact strength observed with glass fiber reinforcement compared with that with TiO₂ nanoparticles alone may be explained by the crack-arresting ability of the fibers. During sudden impact loading, glass fibers absorb and dissipate the fracture energy by bridging the propagating cracks, thereby preventing catastrophic failure. Conversely, TiO₂ nanoparticles primarily strengthen the resin matrix but contribute less effectively to energy absorption during impacts. The hybrid reinforcement benefited from both mechanisms simultaneously, which likely explains the highest impact strength recorded in this study.

Therefore, the combined reinforcement of TiO₂ and glass fiber in heat-cured denture base material or reinforcing glass fiber in heat-cured denture base is the recommended choice for enhancing impact strength and prolonging the lifespan of the prosthesis compared with TiO₂ reinforcement alone. The present findings are also in agreement with those of a systematic review by Bangera et al. [7], who concluded that TiO₂ nanoparticles significantly improve the flexural strength of PMMA when incorporated at low concentrations and with adequate dispersion. Similarly, Somani et al. [14] reported in their meta-analysis that both fiber and nanoparticle reinforcement enhanced denture base strength, although fiber reinforcement generally demonstrated a greater improvement in fracture resistance than did particulate fillers. The present findings support these observations, as glass fiber reinforcement produced greater flexural and impact strengths than TiO₂ nanoparticles alone.

This study investigated the combined effects of these materials. The findings confirmed the anticipated outcome, as the combined effect of TiO₂ and glass fibers demonstrated a statistically significant enhancement in both the flexural and impact strengths of the prosthesis, surpassing the individual reinforcement effects of the materials. The differences between the present findings and previous reports may also be related to differences in resin brands, particle morphology, silanization of glass fibers, specimen storage conditions, thermocycling protocols, polymer-to-monomer ratio, and testing standards (ISO vs. ASTM). These methodological differences can substantially influence the measured mechanical properties and should be considered when comparing the results of different studies.

From a clinical perspective, improved flexural strength may reduce fatigue fractures during mastication, whereas improved impact strength may decrease accidental denture fractures during cleaning or insertion. Therefore, hybrid reinforcement has the potential to enhance prosthesis longevity, reduce repair frequency, and improve patient satisfaction, particularly among elderly patients who are more susceptible to accidental denture drop.

Limitations

The sample size was relatively small, with only five specimens per group for flexural and impact testing, which may limit the statistical power and generalizability of the findings. Being an in vitro investigation, it does not replicate the complex oral environment, including thermal cycling, salivary enzymes, pH fluctuations, and functional chewing forces; hence, the results may not translate directly to clinical performance. Only a single concentration (2 wt%) of TiO₂ nanoparticles and glass fiber was evaluated, without exploring the effect of varying concentrations or fiber lengths and orientations on mechanical strength. Furthermore, the study assessed only flexural and impact strength, while other clinically relevant properties, such as surface hardness, wear resistance, water sorption, solubility, and color stability, were not investigated. The homogeneity of fiber and nanoparticle distributions within the resin matrix was not verified using techniques such as scanning electron microscopy, and manual incorporation may have introduced variability that was not accounted for in the results. Additionally, specimens were not subjected to thermocycling or long-term water storage, both of which are known to influence the mechanical behavior of PMMA-based materials, and only a single standardized testing protocol (ISO #1567) was used, which may limit comparability with studies employing different testing configurations.

## Conclusions

Within the limitations of this in vitro study, hybrid reinforcement of heat-cured PMMA denture base resin with TiO₂ nanoparticles and glass fiber produced the most favorable mechanical performance, with significantly higher flexural and impact strengths than those of conventional PMMA and PMMA reinforced with either TiO₂ nanoparticles or glass fiber alone. Among the individual reinforcements, glass fiber exhibited a better strengthening effect than TiO₂ nanoparticles, indicating its greater contribution to improving the resistance of the denture base resin to functional and accidental stresses. Although the hybrid group demonstrated the best overall results, the values obtained in all the groups remained within clinically desirable limits. Therefore, combined reinforcement with TiO₂ nanoparticles and glass fibers appears to be a promising approach for improving the durability and fracture resistance of PMMA denture bases; however, further studies under clinical conditions are warranted before routine application.
